# A Rare Presentation of Pseudoaneurysm of the Popliteal Artery After Total Knee Replacement: A Case Report and Review of the Literature

**DOI:** 10.7759/cureus.65772

**Published:** 2024-07-30

**Authors:** Naji Alammari, Biju Ananthan, Saad Alotaibi, Yasser Asiri, Mohammed Mohammed, Abdulmalek Almulla

**Affiliations:** 1 Orthopedics, King Fahad Specialist Hospital, Dammam, SAU; 2 Musculoskeletal Radiology, Medical Imaging Services Center, King Fahad Specialist Hospital, Dammam, SAU

**Keywords:** complications, total knee replacement, total knee arthroplasty, pseudoaneurysm, popliteal artery

## Abstract

Total knee replacement (TKR) is a common successful surgery in terms of the outcomes. The common complications of TKR are joint infection, deep venous thrombosis (DVT), wound complication, and postoperative knee instability. Arterial complications are not common. We are presenting a 61-year-old man who underwent left TKR. Upon postoperative regular follow-up, the patient developed symptoms and clinical presentation for DVT. However, initial duplex ultrasonography was negative for DVT. Repeated duplex ultrasonography showed a pseudoaneurysm of the popliteal artery, and the diagnosis was confirmed with computed tomography (CT) angiography. Pseudoaneurysm of the popliteal artery is a rare complication following primary TKR. Our patient underwent endovascular angioplasty and stenting of the pseudoaneurysm of the left popliteal artery. The patient completed three years and six months of follow-up with an uneventful course. We selected to share our experience of this rare case because pseudoaneurysm of the popliteal artery is a rare complication after TKR, which is usually present with symptoms that can mimic DVT, such as acute lower limb swelling, calf muscle pain, and pain with passive ankle dorsiflexion. Duplex ultrasonography is the preferred first diagnostic tool, and CT angiography (CTA) is needed to confirm the diagnosis and to plan treatment. Treatment with endovascular stent proved to be safe and successful with no infection risk or need for modifying rehabilitation protocol after more than three years of follow-up.

## Introduction

Total knee replacement (TKR) is a surgical procedure that replaces an advanced arthritic knee joint with an artificial joint and is performed for patients suffering from debilitating, end-stage arthritic conditions of the knee. This surgery is usually considered when conservative treatments have failed, with around 94%-97% of all primary TKRs indicated due to osteoarthritis [1]. TKR is one of the most successful surgeries performed in orthopedics. Also, surgical outcomes regarding pain relief, ability to do daily activities, range of motion, and quality of life are dramatically improved [2,3].

Common and well-known complications of TKR include joint infection, deep vein thrombosis (DVT), wound complications, and postoperative knee instability [4]. However, arterial complications are uncommon, and the exact frequencies and clinical sequelae are still poorly organized. Vascular injuries are potentially catastrophic, requiring urgent reconstruction with or without fasciotomies, and delays in diagnosis may lead to amputation [5,6]. Most vascular injury during TKR involves the popliteal artery, and less common studies describe injury to the geniculate arteries [7,8].

The popliteal artery injury can be classified based on the anatomical damage and one of two possible causes. One cause can be occlusion of the artery by thrombosis, which can occur due to (a) low blood flow when a tourniquet is used, (b) knee manipulations that result in microtears and endothelial damage, or (c) thermal injury from the bone cement [9]. A second cause can be the popliteal artery’s sharp transection during the bone cuts [10]. In other words, pseudoaneurysm can form directly from a partial tear in the arterial wall or indirectly from mechanical stretching or thermal injury from the bone cement. Often, pseudoaneurysms can be misdiagnosed as DVT [11,12].

In the following section, we report a case of pseudoaneurysm of the popliteal artery after a TKR with unusual presentation, challenging diagnosis, and successful management.

## Case presentation

History

**Figure 1:**
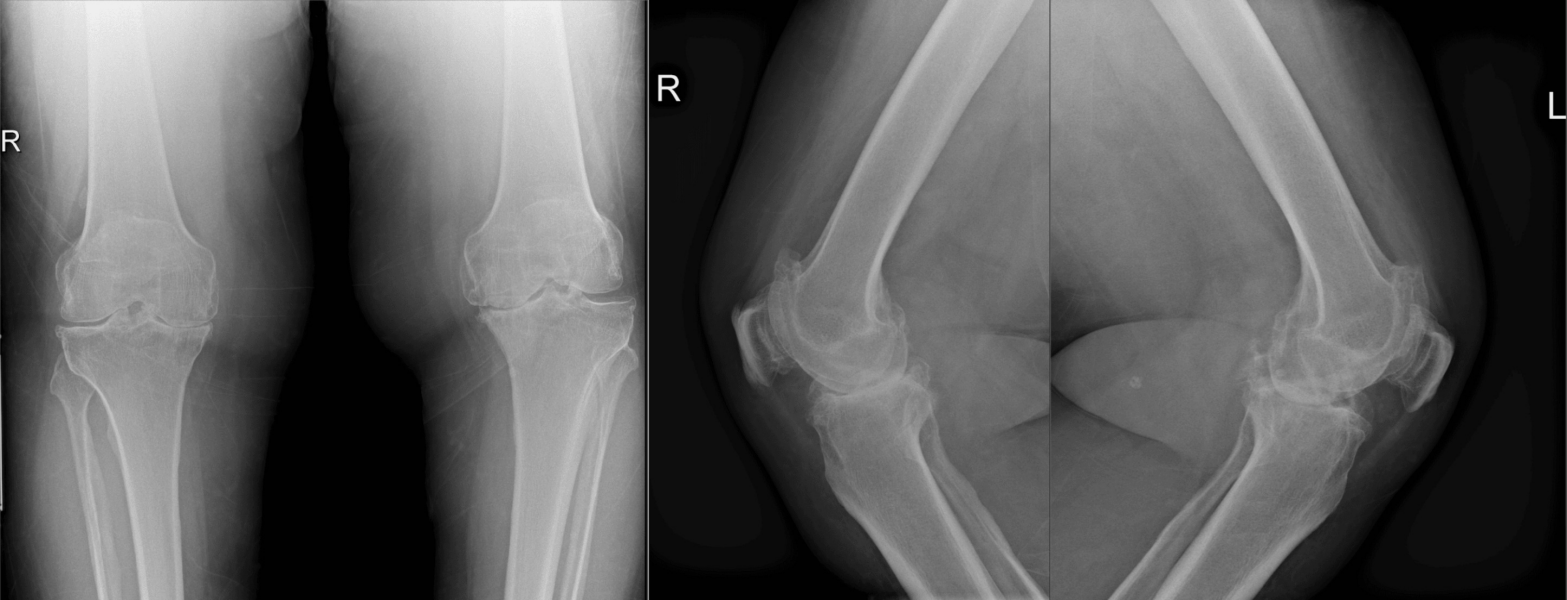
Pre-op x-rays of bilateral knees X-rays of bilateral tri-compartmental advanced osteoarthritic changes in the knees identified medial joint space narrowing, subchondral sclerosis, and osteophyte formation. There were no obvious radiological signs of vascular atherosclerosis.

The patient was a 61-year-old man with a known case of hypertension and type 2 diabetes mellitus, both diagnosed over 10 years ago. He was on oral antihypertensive and hypoglycemic medications. He was followed in an arthroplasty clinic for advanced osteoarthritis in both knees, with more advanced osteoarthritis in the right knee (Figure 1). After conservative management failed, the patient underwent right-side TKR followed by an uneventful postoperative period. In the following year, the pain became more severe on his left side, prompting another TKR.

Surgical technique (left TKR)

We planned for a posterior sacrificing implant from a Smith + Nephew Genesis II posterior stabilized design (PS), an intraoperative course. The patient was in a supine position with a tourniquet applied for 120 minutes. We applied a pneumatic compression device to the right lower limb. We made a midline skin incision and medial para-patellar approach for left TKR after dissecting layer by layer and performing a medial para-patellar arthrotomy. We removed the osteophytes and then released the deep MCL.

After that, we started with a distal femur cut using an oscillating saw with maximum knee flexion. The cut was made based on an intra-medullary guide with a 6-degree angle on the valgus. We then made a tibial cut using an extra-medullary guide. We checked the alignment and balance using the spacer block and rods, and then we completed the femur cut for the anterior, posterior, and chamfers based on the posterior reference. This was done using an oscillating saw with the knee in maximum flexion after ensuring that the soft tissue was protected with a bone Hohmann retractor. We then adjusted the implant for acceptable alignment and balance. We used a cemented PS implant. We removed all the debris and irrigated and closed in layers.

Postoperative hospital course

**Figure 2:**
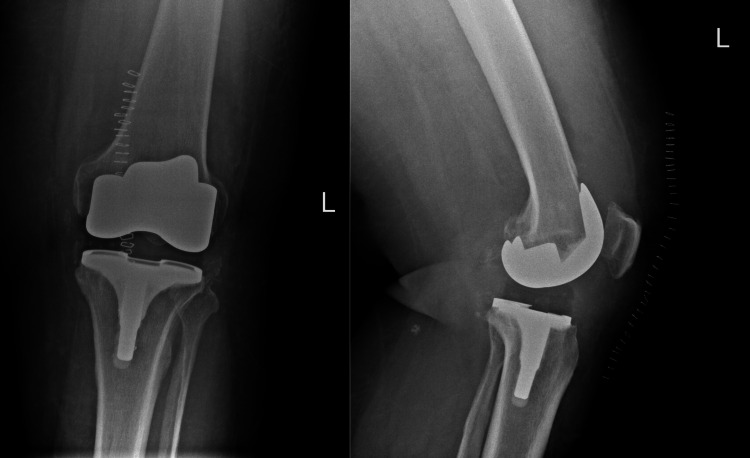
X-rays of post left TKR Status post left knee total replacement shows acceptable implant alignment, size, and position. There are no fractures, translations, or dislocations. Moderate joint effusion is present. Multiple skin staples, soft tissue edema, and air foci are consistent with early postoperative changes. TKR: Total knee replacement.

The patient tolerated the procedure and stayed in the regular ward for three days. The postoperative x-rays were reviewed (Figure 2). During hospitalization, the patient received Cefazolin as post-surgery antibiotics. Also, he received paracetamol and tramadol as post-surgery analgesia with Enoxaparin for DVT prophylaxis. He was discharged on the third day, with the same medications.

Clinical visits

On postoperative day 10, the patient came for wound dressing and complained of left leg pain, numbness, and swelling, mainly on the calf muscle. The swelling increased with passive ankle dorsiflexion, which made us suspect DVT. We conducted duplex ultrasonography of lower limb veins, which was negative for DVT.

On postoperative day 15, the patient arrived at the clinic for skin clip removal. We noticed an increase in thigh girth measurement by about 3 centimeters (cm) and noted the same complaints as before.

**Figure 3:**
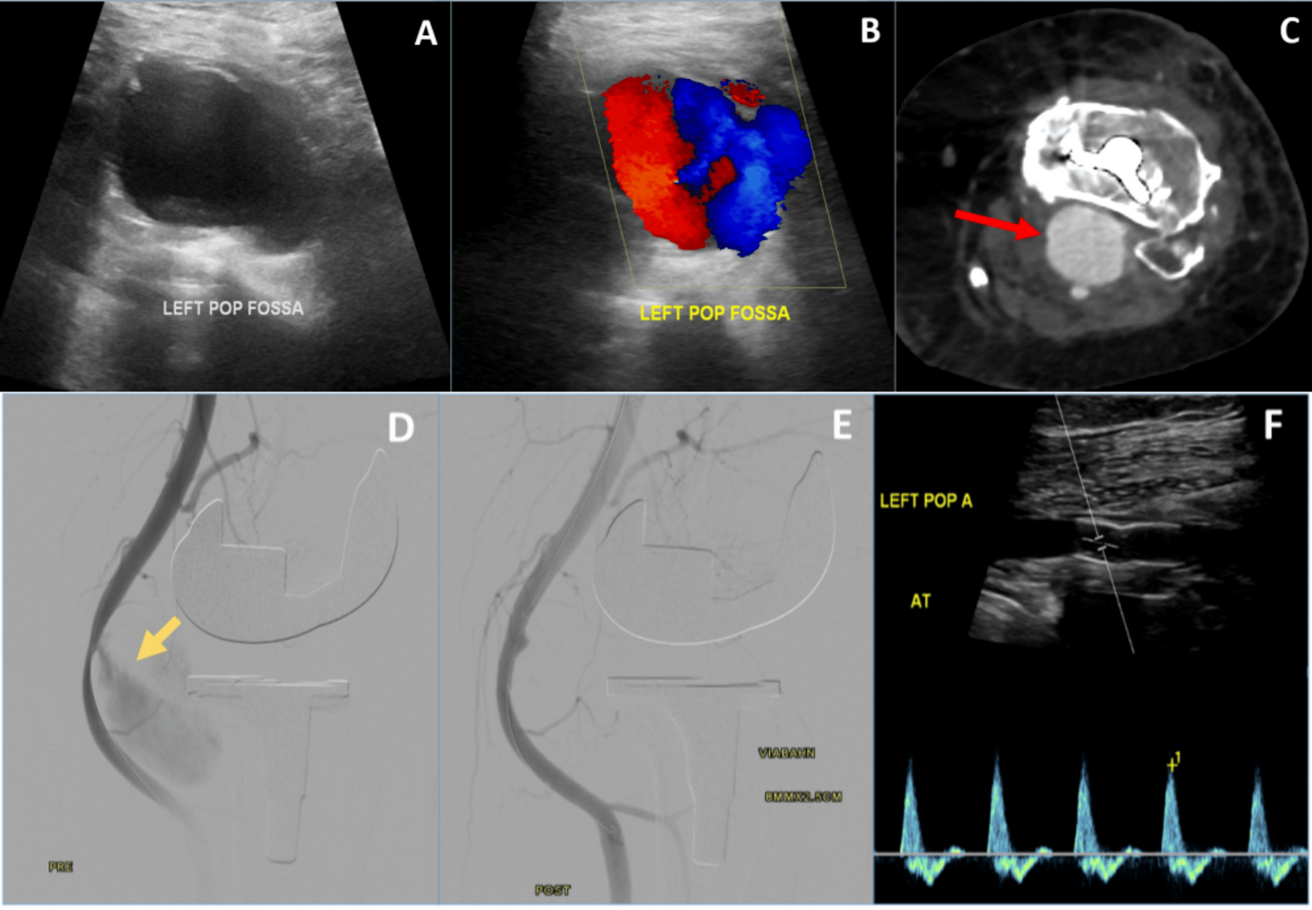
Repeated vascular duplex ultrasonography and angiogram of pre- and post-endovascular angioplasty as well as stenting of the pseudoaneurysm in the left popliteal artery (A) Repeated vascular duplex ultrasonography examination showed a large cyst-like lesion in the left popliteal fossa. (B) Doppler images showed turbulent forward and backward flow within the lesion, giving the classic appearance of a “Yin-Yang” sign. Sonographic findings were consistent with the popliteal artery pseudoaneurysm. (C) The selected axial image at the level of the tibial stem/proximal tibial bone from the CT lower extremity angiogram study confirmed the presence of a large popliteal artery pseudoaneurysm (red arrow). (D) The angiogram of the left lower limb demonstrated a high-flow popliteal pseudoaneurysm (yellow arrow). (E) A covered stent was inserted extravascularly using a flexible soft device to allow excessive movement. Post-stenting angiogram showed successful exclusion of the pseudoaneurysm. (F) Follow-up duplex ultrasonography showed the patient’s popliteal artery with normal flow.

On postoperative day 17, left thigh pain became more disruptive of his daily activities such as walking, sitting, and ground activities; even with anti-inflammatory agents, his pain score was 3/10. He had localized tenderness over the vastus lateralis muscle across the mid-thigh and moderate effusion around the knee without local signs of inflammation. The range of motion of the knee was 0-100 degrees. Distal neurovascular status was intact. There were no clinical signs of DVT, and the wound was healed. However, persistent thigh swelling was noticed as a previous measurement of around 3 cm. So, we decided to repeat the duplex ultrasonography, which showed a 3 cm pseudoaneurysm of a popliteal artery. This was confirmed with computed tomography (CT) angiography (Figure 3).

Treatment planning

We admitted the patient and arranged for a vascular consultation on the same day. We discussed the merits of endovascular repair versus open surgical repair with vascular surgeons and interventional radiologists. The patient consented to both, and, on postoperative day 19, he underwent endovascular angioplasty and stenting of the pseudoaneurysm of the left popliteal artery. The patient resumed anticoagulants in the form of clopidogrel and aspirin 12 hours after the procedure.

**Figure 4:**
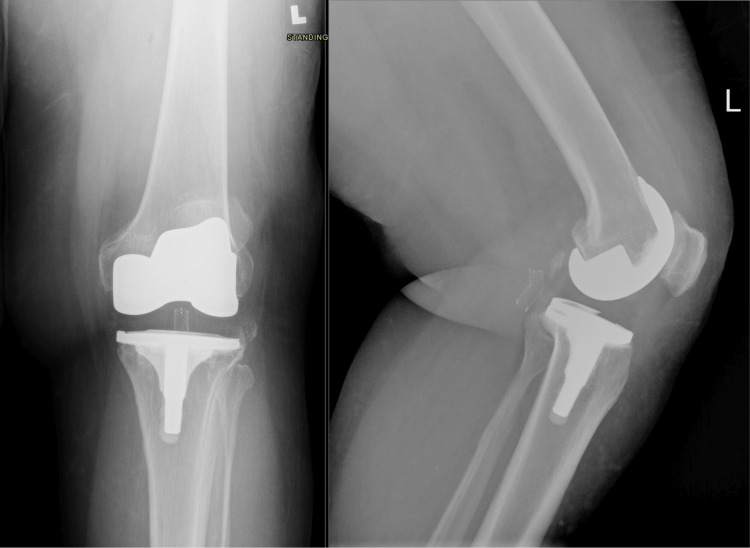
X-rays of left knee after three years and six months of follow-up The left popliteal artery stent is noted. Left total knee replacement with maintained alignment of the prosthesis is observed, and there are no signs of hardware-related complications.

On postoperative day 20, the pain was well controlled, and distal neurovascular examination was intact. We resumed the TKR rehabilitation protocol, and the patient was discharged on postoperative day 21 (Figure 4).

Further follow-up

**Figure 5:**
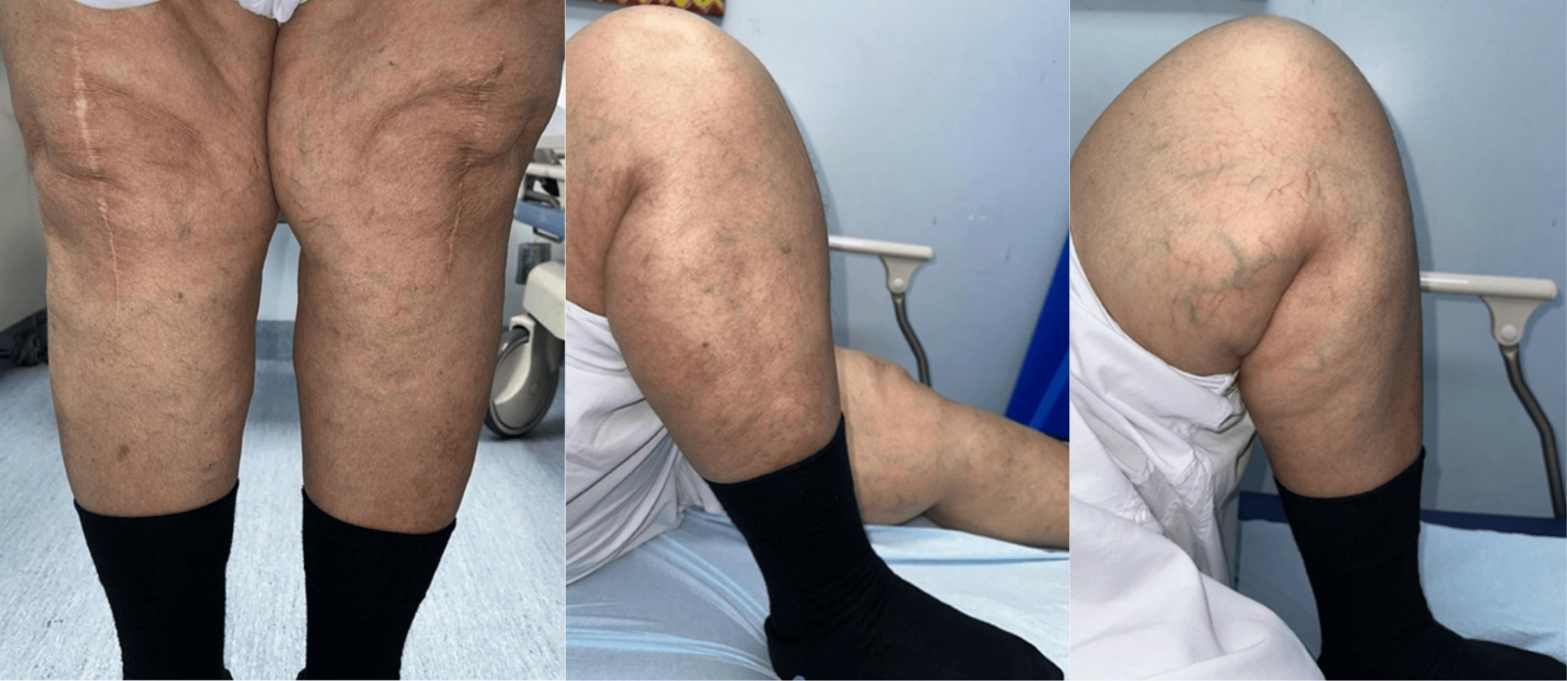
Physical examination of bilateral knees at three years and six months of follow-up The image shows bilateral knees after more than three years, with full extension, flexion greater than 100 degrees bilaterally, and no more swelling in the left lower limb.

The patient was evaluated in the clinic one week after the endovascular stenting of the pseudoaneurysm. He was comfortable, the swelling had subsided, and thigh girth had improved. He had become completely asymptomatic. He was also following the post-TKR rehabilitation protocol. He is still on regular follow-ups with vascular surgery and orthopedic surgery clinics. He has completed three years and six months of follow-up as of now (Figure 5).

## Discussion

Vascular complications after TKR are uncommon, with injuries occurring between 0.03% and 0.2% of cases. Most vascular injuries following TKR result from applying tourniquets over calcified vessels, which can damage the vessels and lead to thrombosis or embolization of calcified plaques. Vascular blunt trauma is more frequently caused by instruments or manipulations, particularly when there are significant soft tissue contractures or a lack of range of motion less than 50 degrees, as in posttraumatic fibrosis [13-15]. In the literature review, pseudoaneurysms following TKR involving the popliteal artery, anterior tibial artery, and geniculate arteries have been reported, with the incidence of pseudoaneurysms of the popliteal artery after TKR increasing from 0.0095% to 0.088% [12,16,17].

Almost all previous reports, as mentioned in Tables 1, 2, described initial clinical presentation in the form of progressive lower limb swelling, posterior knee and calf pain, and tightness of the calf area. The formation of posterior knee hematoma, a pulsatile popliteal fossa mass, and the presence of an audible souffle sound may contribute to the differentiation of clinical presentation between a DVT and a pseudoaneurysm of the popliteal artery. Also, pseudoaneurysms may remain undetected for a long time or present very late [18]. In the case series by Ammori et al., two patients presented initially with compartment syndrome and were treated with fasciotomies before they were diagnosed with popliteal artery pseudoaneurysm [19]. Also, the literature mentioned that the patient can present with recurrent hemarthrosis or neurological symptoms in the form of foot drop or hypoesthesia in the foot, especially over the first web space or on the plantar aspect [18,20,21]. So, DVT is the most likely differential diagnosis with this clinical presentation, especially for lower limb edema, calf pain, and a history of recent surgery. Popliteal artery pseudoaneurysm after TKR is often diagnosed with duplex ultrasonography to rule out DVT. On the other hand, CT angiography (CTA) is the gold standard, which helps to determine the extent of the treatment plan and the type of surgical intervention [22].

**Table 1:** Review and comparison of the different aspects in the literature on similar conditions, such as age, gender, medical history, time when symptoms began, missed diagnoses with DVT, time of diagnosis, chief complaint, and examination TKR: Total knee replacement.

Author, year	Our case, 2024	Padegimas et al., 2016 [30]	Ammori et al., 2016 [19]	Cañibano et al., 2019 [28]	Shin et al., 2014 [21]	Schermer et al., 2022 [31]	Papadopoulos et al., 2015 [32]
Type of study	Case report	Retrospective study. One patient has a popliteal artery pseudoaneurysm.	Retrospective study. 7 patients have popliteal artery pseudoaneurysm.	Case report	Case report	Case report	Case report
Age (years)	61	67	Median 66.5 (57.3–83.2)	76	61	66	76
Gender	Male	Male	Not mentioned	Female	Female	Female	Female
Medical history	Hypertension and diabetes mellitus type II	Diabetes mellitus	Not mentioned	Hypertension	No previous history of arterial or cardiovascular diseases	History of asthma, hypertension, type 2 diabetes mellitus, and obesity	No history of atherosclerotic disease, diabetes, ischemic heart disease, cerebrovascular or peripheral vascular disease
Time of starting symptom	Day 10 post OP	Day 2 post OP	Not mentioned	Day 7 post OP	Day 3 post OP	Day 2 post OP	Day 3 post OP
Missed diagnosis with DVT	No. Was on differential diagnosis only.	No. Was on differential diagnosis only.	No. Was on differential diagnosis only.	No. Was on differential diagnosis only.	No. Was on differential diagnosis only.	No. Was on differential diagnosis only.	Yes
Time of diagnosis	Day 17 post OP	Day 3 post OP	Median 15 (7–27)	Day 7 post OP	Not mentioned	Day 4 post OP	Day 40 post OP
Chief complaint	Left leg pain, numbness, and swelling, mainly in the calf muscle	Pain and edema in the lower limb	Not mentioned	Pain and edema in the lower limb	Right knee and calf swelling, severe pain in the right lower limb, and weakness of right ankle dorsiflexors	Pain in the calf and knee and decreased sensibility of the lateral edge of the foot	Lower limb edema, redness, and pain
Examination	Calf muscle swelling and pain increased with passive ankle dorsiflexion. Localized tenderness over the vastus lateralis muscle over the mid-thigh. Increase in thigh girth measurement by about 3 cm.	Intact distal pulse	Not mentioned	Intact distal pulse and palpable mass in the popliteal area	Intact pulse and weakness of right ankle dorsiflexors. Palpable mass in the popliteal area. Painful palpation of the whole leg.	Intact distal pulse and calf palpation of the whole leg was painful. No neurological deficit.	Intact distal pulse. Palpable mass in the popliteal area.

**Table 2:** Review and comparison of the different aspects in the literature on similar conditions, such as type of TKR, abnormality in duplex ultrasound, diagnostic study, treatment, medications, and outcome TKR: Total knee replacement.

Author, year	Our case, 2024	Padegimas et al., 2016 [30]	Ammori et al., 2016 [19]	Cañibano et al., 2019 [28]	Shin et al,. 2014 [21]	Schermer et al., 2022 [31]	Papadopoulos et al., 2015 [32]
Type of TKR	Primary TKR	Primary TKR	Primary TKR	Primary TKRs	Primary TKR	Revision TKR	Primary TKR
Abnormality in duplex ultrasound	Yes. In second duplex ultrasonography.	Yes	Yes	Yes	No	Yes	Yes
	Day 10 (Negative)						
	Day 17 (Positive)						
Diagnostic study	Duplex ultrasonography and tomography angiography	Duplex ultrasonography	4 patients by duplex ultrasonography, 2 patients by arteriogram,1 patient by computed tomography angiography	Computed tomography angiography	Computed tomography angiography	Computed tomography angiography	Computed tomography angiography
Treatment	Endovascular angioplasty and stenting of the pseudoaneurysm of the left popliteal artery	Stenting of the pseudoaneurysm	For compartment syndrome (6 patients underwent fasciotomies). For popliteal artery pseudoaneurysm (4 patients with posterior operative repair and 2 patients with stent insertion), 1 patient had no intervention because he had a significant reduction in the size of the pseudoaneurysm.	Pseudo-endoaneurysmorrhaphy by lateral suturing of the popliteal artery with monofilament 5/0	Placement of a covered endovascular stent graft	Pseudoaneurysm endovascularly with a covered stent	Surgical exploration of the popliteal artery with the aid of a great saphenous vein patch
Medications	Dual antiplatelet therapy in the form of clopidogrel (3 months) and aspirin (lifelong)	Enoxaparin	Not mentioned	Not mentioned	Not mentioned	Dual antiplatelet therapy for 6 months	Not mentioned
Outcome	Free of symptoms (no pain or swelling)	Free of symptoms (no pain or swelling)	3 patients had neuropathic pain affecting the foot. 1 patient died because of hypertensive heart disease 3 days after the insertion of a stent.	Free of symptoms (no pain or swelling)	Asymptomatic (no pain or swelling). The patient had foot drop (peroneal and posterior tibial nerve dysfunction).	Complaints of hypesthesia of digits 3 to 5 of the right foot and heel due to tibial nerve neuropathy as compressed by pseudoaneurysm	Free of symptoms (no pain or swelling)

Following TKR vascular injuries, above-knee amputations were more common in prior years. However, improvements in diagnostic image modalities and endovascular techniques have decreased the incidence of above-knee amputation [23]. Because of its proximity to the joint, the popliteal artery is most commonly affected. Popliteal artery injury is more common in revision than in primary surgeries because the popliteal artery is hidden by fibrosis during tissue dissection. Rubush et al. explained the jeopardized zone for the popliteal artery during the placement of the posterior retractor, the use of oscillating saw or pins to hold the tibial jig, the posterior capsular release, the posterior cut of the femoral condyles, the application of retractor for anterior dislocation of the tibia, the placement of the knee in hyperextension after the cuts, and before the application of the hardware or tibial cuts. They divide the articular surface of the tibia into a clock, with 6 o’clock being the most anterior position. The jeopardized zone is between 11 and 3 o’clock (12 o’clock is the position of the popliteal vein, 1 o’clock is the position of the popliteal artery, and 2 o’clock is the position of the anterior tibial artery). Popliteal artery trauma in TKR occurs during surgery. It can present as acute ischemia or with arterial thrombosis, pseudoaneurysm, and occlusion [24].

The initial diagnostic tool should be duplex ultrasonography because DVT is the most common differential diagnosis. CTA should be used to confirm the diagnosis and plan treatment. An MRI scan can be performed to confirm the diagnosis as well. However, metallic artifact formation caused by the prosthesis is frequently visible on both CTA and MRI [16,22]. Repeat duplex ultrasonography is also important in determining whether the popliteal artery pseudoaneurysm is increasing in size [25].

The literature reports a remarkably long interval between diagnosis and occurrence of the popliteal artery pseudoaneurysm during surgery. Ammori et al. found a median interval of 15 days (range: 7-27 days) [19]. In the series by Bernhoff et al., a popliteal artery pseudoaneurysm was diagnosed in 11 patients with a median time interval to achieve the final diagnoses of 41 days (range: 2-90 days) [26]. Compared to the literature, our diagnosis was about the same (17 days postoperatively) on second duplex ultrasonography. This second duplex ultrasonography was indicated due to persistent symptoms and to exclude DVT. Also, the patient had an early clinical presentation (day 10 postoperative) because the popliteal artery pseudoaneurysm was identified quickly. The case needs comprehensive and urgent workups such as duplex ultrasonography followed by CTA for confirmation of the diagnosis, followed by vascular surgery and intervention radiology for the planning of treatment.

In the treatment of popliteal pseudoaneurysms, there are several procedures. Vein-bypass open surgery has been used extensively with good long-term outcomes. However, it has a high risk of injury to adjacent structures and prosthesis infection because of the presence of fibrosis and anatomical changes due to previous surgery. Another therapeutic procedure is endovascular treatment with stents, which has frequently been described in the literature as the management of popliteal artery pseudoaneurysms with good results [27,28]. Published results using stents are satisfactory, although in our case, the treatment was endovascular angioplasty and stenting of the pseudoaneurysm in the left popliteal artery. It had a good outcome, and the patient was asymptomatic from day 1 after endovascular angioplasty and stenting with more than three years of follow-up. A less invasive therapeutic procedure is ultrasound-guided compression or thrombin injection to achieve thrombosis. This technique is described in the literature with minimal data showing good results; however, as only a few cases have been reported and data are not still conclusive, its use is not standardized [29-32].

In the case of large pseudoaneurysms such as ours, the vascular surgeon offered an open repair versus endovascular angioplasty and stenting treatment. Ultimately, the vascular surgeon preferred endovascular angioplasty and stenting treatment. For many reasons, open repair presents a unique set of challenges: It requires a posterior knee approach, the presence of fibrosis, and anatomical changes due to previous surgery that increase the possibility of harming nearby structures and putting the newly implanted prosthesis at risk of infection. In addition, it necessitates harvesting a venous graft from the contralateral limb. Despite the lack of long patient series, our understanding is based on data showing that endovascular therapy can be applied with a manageable morbidity rate and is both safe and long-lasting when used to treat traumatic popliteal artery pseudoaneurysms.

## Conclusions

A pseudoaneurysm of the popliteal artery is a rare complication after TKR, which is usually present with symptoms that can mimic DVT, such as acute lower limb swelling, calf muscle pain, and pain with passive ankle dorsiflexion. Duplex ultrasonography is the preferred first diagnostic tool, and CTA is needed to confirm the diagnosis and plan treatment. Treatment with endovascular stent proved to be safe and successful with no infection risk or a need to modify the rehabilitation protocol after more than three years of follow-up.

## References

[1] Van Manen MD, Nace J, Mont MA (2012). Management of primary knee osteoarthritis and indications for total knee arthroplasty for general practitioners. J Am Osteopath Assoc.

[2] Guo B, Qin S, Huang Y (2018). [Research progress of knee-salvage treatment for knee osteoarthritis]. Zhongguo Xiu Fu Chong Jian Wai Ke Za Zhi.

[3] Ghosh A, Chatterji U (2019). An evidence-based review of enhanced recovery after surgery in total knee replacement surgery. J Perioper Pract.

[4] Healy WL, Della Valle CJ, Iorio R, Berend KR, Cushner FD, Dalury DF, Lonner JH (2013). Complications of total knee arthroplasty: standardized list and definitions of the Knee Society. Clin Orthop Relat Res.

[5] McAuley CE, Steed DL, Webster MW (1984). Arterial complications of total knee replacement. Arch Surg.

[6] Stewart AH, Baird RN (2001). The prevention and early recognition of arterial complications in total knee replacement: a vascular surgical perspective. Knee.

[7] Ko LJ, DeHart ML, Yoo JU, Huff TW (2014). Popliteal artery injury associated with total knee arthroplasty: trends, costs and risk factors. J Arthroplasty.

[8] Julien TP, Gravereaux E, Martin S (2012). Superior medial geniculate artery pseudoaneurysm after primary total knee arthroplasty. J Arthroplasty.

[9] Khan S, Salam H, Kessels J (2014). Popliteal artery occlusion after total knee replacement: a vascular team approach for limb salvage. Vasc Dis Manage.

[10] Saksena J, Platts AD, Dowd GS (2010). Recurrent haemarthrosis following total knee replacement. Knee.

[11] Choksey A, Noble J, Brown JJK, Marcuson RW (1998). Angiography in vascular problems with total knee replacement: a report of three cases. Knee.

[12] O'Connor JV, Stocks G, Crabtree JD Jr, Galasso P, Wallsh E (1998). Popliteal pseudoaneurysm following total knee arthroplasty. J Arthroplasty.

[13] Da Silva MS, Sobel M (2003). Popliteal vascular injury during total knee arthroplasty. J Surg Res.

[14] Smith DE, McGraw RW, Taylor DC, Masri BA (2001). Arterial complications and total knee arthroplasty. J Am Acad Orthop Surg.

[15] Kerens B, Boonen B, Schotanus MG, Kort NP (2013). Popliteal lesion due to traction during unicompartmental knee revision surgery. J Orthop.

[16] Calligaro KD, Dougherty MJ, Ryan S, Booth RE (2003). Acute arterial complications associated with total hip and knee arthroplasty. J Vasc Surg.

[17] Reynolds A, Sandstrom A, Jha PK (2017). Totally endovascular management of popliteal artery occlusion and pseudoaneurysm formation after total knee replacement. Ann Vasc Surg.

[18] Geertsema D, Defoort KC, van Hellemondt GG (2012). Popliteal pseudoaneurysm after total knee arthroplasty: a report of 3 cases. J Arthroplasty.

[19] Ammori MB, Evans AR, Mclain AD (2016). Popliteal artery pseudoaneurysm after total knee arthroplasty. J Arthroplasty.

[20] Boutchichi A, Ciornohac J, Daubresse F (2013). Pseudoaneurysm after total knee arthroplasty: a rare complication with different possible clinical presentations. Acta Orthop Belg.

[21] Shin YS, Hwang YG, Savale AP, Han SB (2014). Popliteal artery pseudoaneurysm following primary total knee arthroplasty. Knee Surg Relat Res.

[22] Daniels SP, Sneag DB, Berkowitz JL, Trost D, Endo Y (2019). Pseudoaneurysm after total knee arthroplasty: imaging findings in 7 patients. Skeletal Radiol.

[23] Rand JA (1987). Vascular complications of total knee arthroplasty. Report of three cases. J Arthroplasty.

[24] Rubash HE, Berger RA, Britton CA, Nettrour WS, Seel MJ (1993). Avoiding neurologic and vascular injuries with screw fixation of the tibial component in total knee arthroplasty. Clin Orthop Relat Res.

[25] Mofidi R, Kelman J, Berry O, Bennett S, Murie JA, Dawson AR (2007). Significance of the early postoperative duplex result in infrainguinal vein bypass surveillance. Eur J Vasc Endovasc Surg.

[26] Bernhoff K, Rudström H, Gedeborg R, Björck M (2013). Popliteal artery injury during knee replacement: a population-based nationwide study. Bone Joint J.

[27] Troutman DA, Dougherty MJ, Spivack AI, Calligaro KD (2013). Updated strategies to treat acute arterial complications associated with total knee and hip arthroplasty. J Vasc Surg.

[28] Cañibano EB, Fresnillo BG, Requena MG (2008). Pseudoaneurysm of the popliteal artery as a complication of knee prosthesis surgery: Endovascular treatment. Angiología.

[29] Arquillo IL, Ferreiroa CG, Muñoz EF, Rey JV, Gómez TB (2011). Poptileal artery complications after total knee arthroplasty. Angiology.

[30] Padegimas EM, Levicoff EA, McGinley AD, Sharkey PF, Good RP (2016). Vascular complications after total knee arthroplasty-a single institutional experience. J Arthroplasty.

[31] Schermer BA, Berger AC, Stomp W, van der Lugt JC (2022). Pseudoaneurysm of the popliteal artery after (revision) knee arthroplasty. Arthroplast Today.

[32] Papadopoulos DV, Koulouvaris P, Lykissas MG, Giannoulis D, Georgios A, Mavrodontidis A (2015). Popliteal artery damage during total knee arthroplasty. Arthroplast Today.

